# Utility of Point of Care Ultrasound in Diagnosis of CRAO

**DOI:** 10.51894/001c.144544

**Published:** 2025-09-30

**Authors:** Bryan Kowalkowski

**Affiliations:** 1 Department of Emergency Medicine Henry Ford Jackson

## 95

### INTRODUCTION

Central Retinal Artery Occlusion (CRAO) is an emergent, time-sensitive, vision-threatening disease requiring prompt diagnosis and treatment. The diagnosis is made largely through history and physical exam, relying heavily on the fundoscopic exam which can be limited in the Emergency Department setting. Point-of-care ultrasound (POCUS) is a rapid, non-invasive modality to evaluate for acute ocular pathology, including CRAO. Here, we present a case of CRAO in a patient with a limited fundoscopic exam diagnosed with POCUS and complicated by GCA.

### CASE DESCRIPTION

A 75-year-old male with a past medical history of hypertension, hyperlipidemia, alcohol use disorder presented to the emergency department with a chief complaint of sudden onset, painless vision loss of right eye. Work up in the ED included CT scan of the head and CTA of Head/Neck. Results of CT imaging were negative for acute pathology. Ocular POCUS was performed and revealed a hyperechoic lesion where central retinal artery entered eye. These findings were consistent with retrobulbar spot sign. Blood work was notable for elevated inflammatory markers including ESR >140 and CRP 2.5 which raised our suspicion for arteritic CRAO.

### DISCUSSION/CONCLUSION

CRAO is classically diagnosed by dilated fundoscopic exam. Findings on exam include a cherry red fovea, pale retina, and box-carring of retinal vessels. Ocular ultrasound can aide in the diagnosis, especially if fundoscopic exam is limited.

Our patient was transferred to a tertiary care facility for ophthalmology evaluation and further stroke workup. Dilated fundoscopic exam by ophthalmology was limited due to large cataract in patient’s right eye. Fluorescein angiography was completed with limited results. Patient was treated with IV steroids during inpatient workup. Temporal artery biopsy and ultrasound were completed but did not show any evidence of active arteritis. Patient was eventually discharged with steroid taper and started on 81 mg daily aspirin. CRAO remained leading diagnosis.

